# A study on the correlation between radiation field size and gamma index passing rate for MatriXX

**DOI:** 10.1097/MD.0000000000016536

**Published:** 2019-07-26

**Authors:** Kai Xie, Hongfei Sun, Liugang Gao, Jianfeng Sui, Tao Lin, Xinye Ni

**Affiliations:** a Department of Radiation Oncology, Changzhou No. 2 People's Hospital, Nanjing Medical University; b The Center for Medical Physics of Nanjing Medical University, Changzhou, China.

**Keywords:** field size, gamma index passing rate, MatriXX, passing rate, quality assurance

## Abstract

This study aimed to analyze the influence of the radiation field size on the passing rate of the treatment planning system using MatriXX if the field irradiated the circuit.

Two sets of static fields which were 10 cm and 30 cm in the left-right direction (X), and was 31 cm to 40 cm in gun-target direction (Y) were designed. In these fields, the gantry was 0 and the monitor units were 200 MU. Two plans from an esophagus carcinoma patient with a planning target volume of 86.4 cm^3^ and a cervical carcinoma patient with a planning target volume (PTV) of 2094.1 cm^3^ were chosen. The passing rates of these plans were gained without and with protecting the circuit area from lead alloys. The gamma analysis was used and the standard was set to 3%/3 mm.

The verification passing rate decreased from 95.0% to 69.2% when X was 10 cm while Y increased from 31 cm to 40 cm. With the protection from low melting point lead alloys, the passing rate was from 96.2% to 89.6%. The results of the second set of plans without lead alloys were similar but the passing rate decreased more sharply. The passing rates of the 2 patients were 99.5% and 57.1%. With the protection of the lead alloys, their passing rates were 99.8% and 72.1%, respectively.

The results showed that with the increase of the radiation field size in the Y direction, more areas were irradiated in the circuit, and the passing rate gradually decreases and dropped sharply at a certain threshold. After putting lead alloys above the circuit, the passing rate was much better in the static field but was still less than 90% in the second patient volumetric modulated arc therapy (VMAT) because the circuit was irradiate in other directions. In daily QA, we should pay attention to these patients with long size tumor.

## 1 Introduction

With the development of precise radiotherapy technology, volumetric modulated arc therapy (VMAT), have been extensively applied. Generally, VMAT is modulated by many computer-controlled multileaf collimators (MLC), which divides into subsegments that are irregular in shape and position. Such complicated beam delivery in VMAT brings a need for thorough quality assurance (QA).^[1]^ It requires high-quality QA tools and radiotherapy equipment to ensure the reliability QA results.^[2]^

Common equipment for patient-specific QA includes photographic films^[3,4]^ and electronic portal imaging devices^[5,6]^. In the past few years, 2D arrays of electronic detectors have become popular.^[7,8]^ The MatriXX ionization chamber (IBA Dosimetry, GmbH, Schwarzenbrook, Germany), a 2D planar dose measurement and verification tool, is used to conduct QA work .^[9–11]^ It can conveniently acquire point dose and 2D dose distributions in the execution plan^[12]^. It also has an angular correction detecting instrument, which will record angle values at an appropriate time and apply the corresponding angular correction factor to the measurement result.

Large-zone disease entities, such as cervical carcinoma and rectal carcinoma, are abundant among radiotherapy patients, and the planning target volume (PTV) size in the gun-to-target (G-T) direction can reach as long as 40 cm. The effective detection areas of commonly used verification equipment, such as MapCheck and MatriXX, are 22 cm × 22 cm and 24.4 cm × 24.4 cm, respectively. And the effective detection areas of EPIDs from several manufacturers are 30 cm × 30 cm.^[13]^ All these values cannot completely cover a radiation field like 40 cm × 40 cm.

To date, many comprehensive studies have been conducted on MatriXX like energy response and angular response^[14–16]^. However, most literature about the influence of the radiation field size on the passing rate is limited to the effective measurement range of MatriXX.^[17,18]^

In routine VMAT QA using MatriXX, we found the passing rates in some patients whose PTVs were long in the G-T direction were below than 90%, even only 60%. To investigate the influence of the radiation field size on the passing rate, a series of radiation fields with different sizes was designed to provide assistance in achieving correct clinical applications. The gamma analysis method with a standard of 3 mm/3% was used in this study, and the passing rate was qualified if it reached 90%.^[19–21]^

## 2 Methods and materials

### 2.1 Equipment

The linear accelerator used was Infinity from Elekta Corporation (Elekta, Stockholm, Sweden). Its MLC had 80 pairs of leaves, and the width of each leaf was 5 mm. X-ray energy was 6 MV. The TPS used was Elekta's Monaco (version no. 5.11.01), the algorithm was the Monte Carlo calculation, the computational grid was 0.3 cm, and uncertainty was 1%. The equipment was MatriXX Evolution from IBA Corporation (Scanditronix Wellhofer GmbH, Germany), which has 1020 ion chambers 4.5 mm diameter × 5 mm height, with 7.62 mm spacing. The analysis software used was My QA (version 2.6.10.0) from IBA Corporation.

### 2.2 Treatment plans design

**Figure 1:**
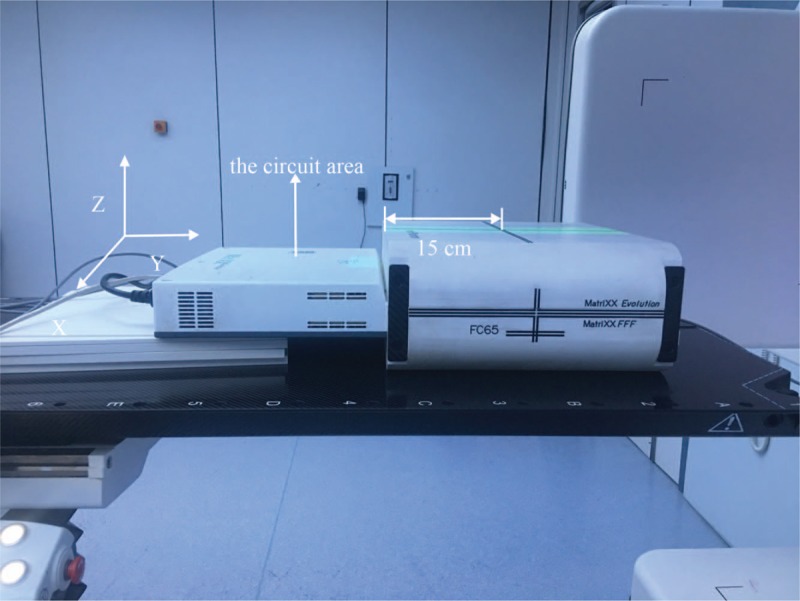
MatriXX placed on the treatment and the orientation used for measurements.

The field was long in the G-T direction where its passing rates were affected and the circuit area of MatriXX was irradiated. A series of static field plans with different size were designed and 2 VMAT plans were chosen as below. The left-to-right direction was set as the X direction, and the G-T direction was set as the Y direction. MatriXX was arranged as shown in Fig. 1.

**Figure 2:**
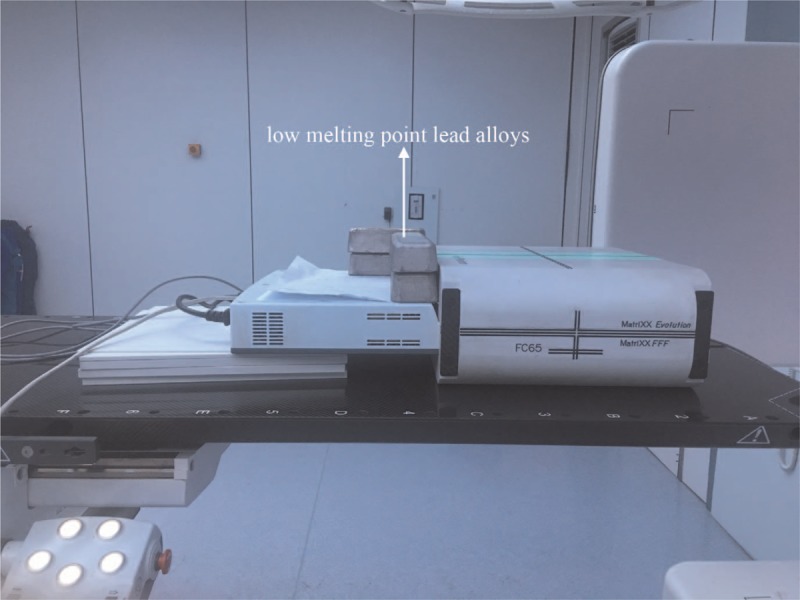
The circuit area protected by low melting point lead alloys.

(1) Static fields which were 10 cm in the X direction and were from 31 cm to 40 in the Y direction were measured. The gantry angle was 0, the monitor units were 200 MU, and the dose rate was 600 MU/min.
(2) Similarly, static fields which were 30 cm in the X direction were measured.
(3) Two patient VMAT plans were selected. The first patient was esophagus cancer, with PTV 5.8 cm, 7.2 cm, 8.7 cm in the X, Y, Z direction. The other patient was cervical carcinoma, with PTV 14.5 cm, 39 cm, 12.1 cm, in the X, Y, Z direction.
  Background recalibration was performed before each set to get accurate results.
(4) After adding 7 cm thick low melting point lead alloys to protect the circuit area as shown in Fig. 2, repeat the previous measurement.
(5) The background doses during 120 seconds were measured before and after the circuit was irradiated.

The present study was approved by the Medical Ethics Committee of The Second People's Hospital of Changzhou (Changzhou, China), and all patients were provided written informed consent for participation.

## 3 Results

**Figure 3:**
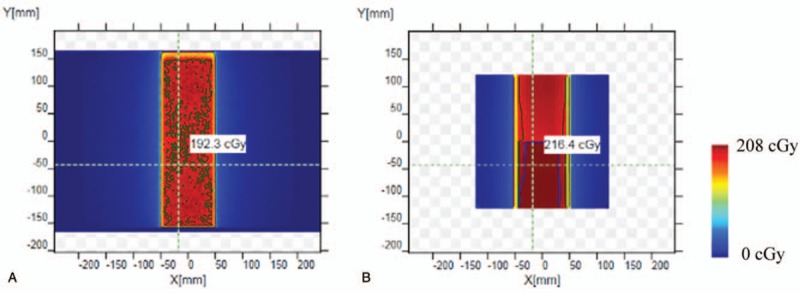
The dose distribution comparison in 10 cm × 40 cm field. (a) Dose distribution obtained in TPS. (b) Dose distribution obtained through measurement.

As shown in Table 1, the verification passing rate decreased from 95.0% to 69.2% when field size was 10 cm in the X direction while field size increased from 31 cm to 40 cm in the Y direction, and changed quickly when field size was near 40 cm. With the protection from low melting point lead alloys, the passing rate was from 96.2% to 89.6%. When the field size was 30 cm in the X direction, the results without lead alloys were similar but the passing rate decreased more sharply (Table 2). Fig. 3 showed a comparison between the calculated and measured dose distributions in 10 cm × 40 cm field. Dose difference was mainly near the circuit area, and the measured values were larger.

**Table 1:**
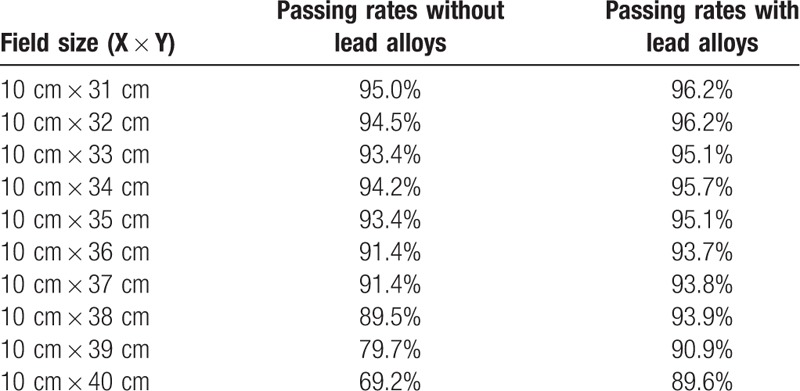
The passing rates in the first set fields without and with lead alloys.

**Table 2:**
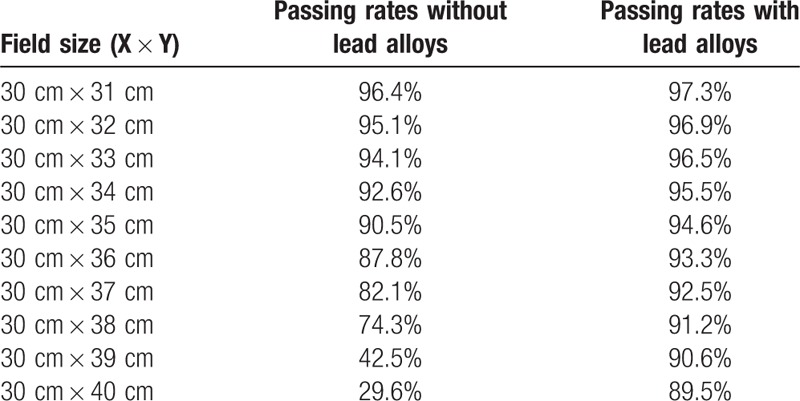
The passing rates in the second set fields without and with lead alloys.

**Figure 4:**
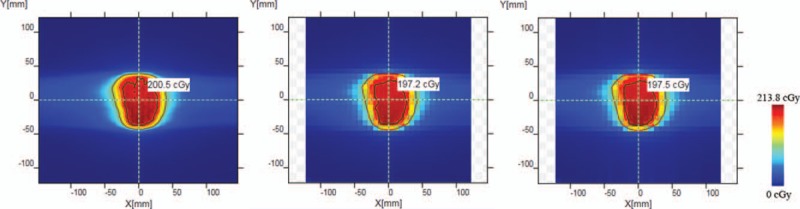
The dose distribution comparison in the first patient. (a) Dose distribution obtained in TPS. (b) Dose distribution obtained through measurement without lead alloys. (c) Dose distribution obtained through measurement with lead alloys.

**Figure 5:**
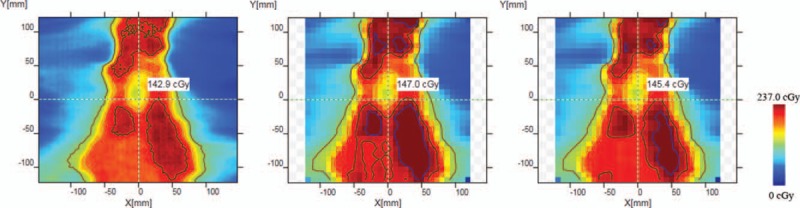
The dose distribution comparison in the second patient. (a) Dose distribution obtained in TPS. (b) Dose distribution obtained through measurement without lead alloys. (c) Dose distribution obtained through measurement with lead alloys.

Figs. 4 and 5 have shown the passing rate results of 2 patients from TPS calculation, measurement without and with lead alloys. The results without lead alloys were 99.5% and 57.1%, respectively. With the protection from lead alloys, the passing rates were 99.8% and 72.1%, respectively.

**Figure 6:**
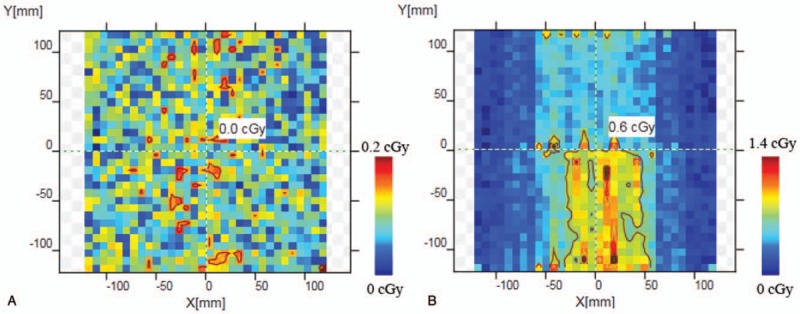
The background doses during 120 seconds before and after the circuit was irradiated. (a) Before the circuit was irradiated. (b) After the circuit was irradiated.

The background doses during 120 seconds were measured before and after the circuit was irradiated, and the results were shown as Fig. 6. The maximum dose was 0.2 cGy normally and could be up to 1.4 cGy abnormally.

## 4 Discussion

In the first set of fields, the passing rates abruptly decreased when field size reached 38 cm in the Y direction, and the rate of 10 cm × 40 cm field was only 69.2% without protection from lead alloys. When X was 30 cm, the passing rates decreased more quickly. The circuit area edge was 15 cm from the center in Fig. 1. The field larger than 30 cm in the Y direction would irradiate the circuit area. As the size increased, so did the exposure area. We could draw the conclusion that the passing rate was affected by the irradiated circuit area.

Fig. 3 showed that the dose distribution in 10 cm × 31 cm field was uniform. In contrast, the dose distribution in 10 cm × 38 cm field could be divided into 2 parts, and the dose near the circuit was significantly higher than that on the other side. With the protection from lead alloys, the circuit area was irradiated much less, and the passing rate was much higher.

At the same time, Fig. 6 showed that the accuracy of dosimeter measurement was affected after the circuit was irradiated. In Fig. 5, the passing rate in the second patient was still less than 90%, this was mainly because the plan was VMAT, and the circuit was irradiate in other directions.

The verification passing rate of the VMAT in the first patient was slightly higher than those of the static fields because a 5% threshold was set in the gamma analysis.^[22]^ The results of Wagner et al indicated that the verification passing rates of radiation fields using MatriXX between 7 cm × 7 cm and 24 cm × 24 cm could reach 99%, whereas the verification passing rate under the 30 cm × 30 cm condition was 82.2%.^[23]^ The average gamma pass was 96.7% ± 2.2% for VMAT in the results of Sanghangthum et al^[24]^ and was similar to our results with lead alloys.

We hold the opinion that when the circuit area was irradiated, it affected the accuracy of the detectors, especially near the circuit area in the Y direction. This is the case with large pelvic, abdomen, hemi-body or whole body QA. With the help of lead alloys, the passing rates of the static field and VMAT could be better. However, due to the thickness and only 1 side of the lead alloys, the results were still affected.

There were limitations in the present study. First, we could not make lead alloys that could protect the circuit in all direction. Second, long time circuit irradiation might affect the MatriXX service life. At last, more MatriXXes from different hospitals could be tested to gain a more reliable result.

## 5 Conclusions

MatriXX plays an important role in daily dose validation. We designed a series of plans to verify the relationship between the passing rate and the field size if the field irradiated the circuit. This study showed that with the increase of the radiation field size in the Y direction, more areas were irradiated in the circuit, and the passing rate gradually decreases and dropped sharply at a certain threshold. After putting about 7 cm thick lead alloys above the circuit, the passing rate increased a lot in static fields. In daily QA, we should pay attention to the irradiation of the circuit area and take reasonable measures to solve it.

## Author contributions

**Conceptualization:** Kai Xie, Xinye Ni.

**Data curation:** Kai Xie, Liugang Gao, Jianfeng Sui.

**Funding acquisition:** Xinye Ni, Liugang Gao.

**Methodology:** Hongfei Sun, Jianfeng Sui.

**Software:** Hongfei Sun, Tao Lin.

**Writing – original draft:** Kai Xie.

**Writing – review & editing:** Xinye Ni.
